# Engaging primary care professionals in OECD’s international PaRIS survey: a documentary analysis

**DOI:** 10.1186/s12961-024-01170-2

**Published:** 2024-07-04

**Authors:** Candan Kendir, Michael van den Berg, Janika Bloemeke-Cammin, Oliver Groene, Frederico Guanais, Andree Rochfort, Jose M. Valderas, Niek Klazinga

**Affiliations:** 1 Directorate for Employment, Labour and Social Affairs, Organisation for Economic Co-Operation and Development (OECD), Paris, France; 2 Department of Public and Occupational Health, Amsterdam University Medical Centres (UMC), Amsterdam, Netherlands; 3 Research & Innovation, OptiMedis, Hamburg, Germany; 4 Faculty of Management, Economics and Society, University of Witten/Herdecke, Witten, Germany; 5 Quality Improvement, Irish College of General Practitioners, Dublin, Ireland; 6 Department of General Practice and Forensic & Legal Medicine, School of Medicine, University College Dublin, Dublin, Ireland; 7 Department of Family Medicine, National University Health System, Singapore, Singapore; 8 Centre for Research in Health Systems Performance (CRiHSP), Yong Loo Lin School of Medicine, National University of Singapore, Singapore, Singapore

**Keywords:** Primary care, Quality of care, Health research, Health policy, Stakeholder engagement, Meaningful engagement, Family doctor, General practitioner, Patient-reported outcome measures, Patient-reported experience measures

## Abstract

Healthcare professionals have first-hand experience with patients in clinical practice and the dynamics in the healthcare system, which can be of great value in the design, implementation, data analysis and dissemination of research study results. Primary care professionals are particularly important as they provide first contact, accessible, coordinated, comprehensive and continuous people-focused care. However, in-depth examination of the engagement of health professionals in health system research and planning activities—how professionals are engaged and how this varies across national contexts- is limited, particularly in international initiatives. There is a need to identify gaps in the planning of engagement activities to inform the design and successful implementation of future international efforts to improve the responsiveness of health systems to the changing needs of patients and professionals. The aim of this study was to explore how primary care professionals were engaged in the design and implementation plans of an international health policy study led by the Organisation for Economic Co-operation and Development (OECD). The OECD’s international PaRIS survey measures and disseminates information on patient-reported outcome and experience measures (PROMs and PREMs) of people living with chronic conditions who are managed in primary care. A documentary analysis of 17 written national implementation plans (country roadmaps) was conducted between January and June 2023. Two reviewers independently performed the screening and data abstraction and resolved disagreements by discussion. We reported the intended target primary care professionals, phase of the study, channel of engagement, level of engagement, and purpose of engagement. All 17 countries aimed to engage primary care professionals in the execution plans for the international PaRIS survey. While organisations of primary care professionals, particularly of family doctors, were the most commonly targeted group, variation was found in the timing of engagement activities during the different phases of the study and in the level of engagement, ranging from co-development (half of the countries co-developed the survey together with primary care professionals) to one-off consultations with whom. International guidance facilitated the participation of primary care professionals. Continuous collaborative efforts at the international and national levels can foster a culture of engagement with primary care organisations and individual professionals and enhance meaningful engagement of primary care professionals.

**Supplementary Information:**

The online version contains supplementary material available at 10.1186/s12961-024-01170-2.

## Background

Engaging stakeholders, such as primary care professionals, academics and their organisations and bringing them together with health system planners, policy makers and national and international health organisations and meaningfully involving their perspectives is essential to bridge the gap between health research, practice, and policy [1]. Stakeholder engagement can improve the value, trust, relevance, actionability, and impact of research and policy decisions [2]. Healthcare professionals have first-hand experience with patients, clinical practice and the healthcare system, and this experience can be of great value in the study design, implementation, data analysis and dissemination of study findings [3]. Primary care professionals are crucial partners to engage, as their participation is essential for any learning health system.

Healthcare professionals can play different roles in health policy research: as informants, partners, and end-users of findings [4, 5]. They can simply interact with the study team in relation to specific questions, for example, by responding to a survey. They can further take a more active role, such as being part of research networks, advisory boards or research teams. Finally, they can use data and evidence from research to improve their practice. The engagement of healthcare professionals in research can enhance quality improvement by increasing their feelings of ownership of the study and facilitating the integration of evidence-based practices and evidence-informed policy [3–7]. In other words, it can improve the efficiency and impact of research by improving the dissemination and implementation of results [3].

An increasing number of funding agencies have made stakeholder engagement a requirement for research studies [5, 8]. Different aspects of the contextualization of healthcare professional engagement, such as type, stage and level of engagement, facilitators and barriers and impact, have been documented in the literature [3, 5, 9]. A review of pilot projects of the Patient-Centered Outcomes Research Institute (PCORI) found that 87% of researchers reported engaging clinicians [5] and that the main reasons for engaging clinicians were to benefit from their expertise, to obtain clinician buy-in and to ensure more meaningful results. Among these projects, while the majority reported engaging stakeholders as consultants or collaborators, less than 10% reported co-development.

Engaging healthcare professionals can be challenging to put into practice. On the researcher side, barriers in the literature include lack of funding, lack of human resources, difficulties in maintaining ongoing discussions, and finding the balance between academic rigor and incorporating stakeholders’ input [8]. On the provider end, multiple competing priorities, such as patient care, might limit their engagement. Lack of time, lack of organisational or managerial support, and lack of resources are commonly reported barriers [4].

Despite widespread agreement on the importance of meaningful engagement of healthcare professionals in health policy and research, achieving this seems to be challenging. We know relatively little about how the engagement of primary care professionals is implemented in such studies and policy. The information is even scarcer regarding implementation in the context of international studies. There is a need to identify the contextualization of engagement activities—how primary care professionals are engaged and how this varies across national contexts. Empirical evidence from large-scale initiatives would support the design and successful implementation of future international studies to inform health policy.

### PaRIS international survey of people living with chronic conditions

The Organisation for Economic Co-operation and Development’s (OECD) Patient-Reported Indicator Surveys (PaRIS) international survey of people living with chronic conditions is an international study assessing patient-reported outcomes and experiences with primary care [10]. The PaRIS survey aims to provide policy recommendations to improve the quality of primary care as measured from the patient’s perspective. The PaRIS survey is designed and implemented by the OECD member states through a formal affiliated body of the organisation’s Health Committee, called “Working Party for PaRIS” with the assistance of the OECD Secretariat and methodological support from a contracted research team, the PaRIS-SUR Consortium (henceforth: ‘the consortium’ [10]. The governance structure of the PaRIS survey is described elsewhere [10].

At the time of writing, the PaRIS survey has completed data collection for the first cycle of data collection, from over 1600 primary care professionals and 100 000 patients aged 45 years and older in 19 countries. Patients are sampled in the participating countries through their primary care professionals. The primary care facility is the unit of sampling, allowing for the interpretation of PROMs and PREMs in the context of the provision of primary care. The PaRIS survey consists of three phases: a design and development phase (2017–2020), a field trial (2021–2022), and a main survey (2023–2024) [10]. The PaRIS measurement tools (questionnaires) were initially developed in 2020 based on the PaRIS conceptual framework with the involvement of various stakeholders, such as patients, healthcare professionals and policymakers [11]. The measurement tools consist of two instruments: a patient questionnaire (administered online with alternative modes of data collection such as paper-and-pencil and telephone) and a provider questionnaire (administered online). In 2021–2022, 17 countries field-tested the study design and PaRIS questionnaires, which were subsequently adapted with further input from stakeholders.

### Engaging primary care professionals in the development of the PaRIS survey

Patients and primary care professionals participated in the design and development of the PaRIS survey at the international level [11]. For example, members of the PaRIS Patient Advisory Panel participated in a modified Delphi process to develop the PaRIS patient questionnaire. Representatives from the World Organisation of Family Doctors (WONCA) advised on which chronic conditions to include and how to capture primary care issues in the PaRIS instruments. Early findings from the Field Trial of the PaRIS survey showed that stakeholder engagement and adaptation to national contexts were the two major stakes in striking the balance between scientific rigor and practical implementation of the PaRIS survey. Therefore, to ensure that the PaRIS survey results are relevant at the national level and can be translated into health policy, it is essential that primary care professionals are closely involved in the implementation of the survey in participating countries.

### This study

This study aims to analyse engagement activities with primary care professionals in the implementation of an international survey. The research question that guided this study was how primary care professionals were planned to be engaged in the execution of the PaRIS survey in 17 countries. The specific questions were as follows:

- Why do project managers plan to engage with primary care professionals?
- Who is the intended target group of primary care professionals?
- When do project managers plan to engage primary care professionals?
- What are the engagement activities that project managers plan to use?
- How does the engagement level and planned activities vary across countries?
- What are the anticipated challenges and facilitators for engagement?

The results of this study will contribute to the literature by sharing concrete examples of how primary care professionals can be engaged in participating countries in the context of international studies. The documentation of various aspects of engagement can accelerate the engagement of primary care professionals in health research and policy in the future.

## Methods

To answer the research questions, a documentary analysis of written national implementation plans (country roadmaps) of 17 participating countries was carried out between January and June 2023. No ethical approval was needed for the study, as it does not include any data from human participants.

### Data/data sources

#### Characteristics of country roadmaps

Based on the international PaRIS guidelines [10], a country roadmap consists of seven main sections: summary, survey introduction and background, sampling, mode and strategy for data collection, ethics, data management and governance, and communication, engagement and dissemination. Country roadmaps are comprehensive and detailed plans that outline all the steps that needed to be taken for successful implementation of the survey in each country. An average country roadmap is 15–20 pages long, and all country roadmaps are written in English.

For the implementation of the PaRIS survey, 17 countries (Australia, Belgium, Canada, Czechia, France, Greece, Iceland, Italy, Luxembourg, Netherlands, Norway, Portugal, Romania, Saudi Arabia, Slovenia, Spain, Wales (UK)) completed their country roadmaps between January and June 2023 and planned to collect data from primary care professionals in 2023. Two countries (Israel and Switzerland) had extended timelines to implement the study and were excluded from this study. United States implemented PaRIS modules into a broader national ongoing initiative (Medicare Current Beneficiary Survey [12]) concerning patient outcomes and experiences and did not collect data from primary care professionals. Therefore, this country was also excluded from this study.

#### Development of country roadmaps

National project management teams serve as the primary national contact points, liaising with the consortium and the OECD on all issues related to the execution of the PaRIS survey. National project management teams were assigned by the country officials and were expected to have some general and specific competencies as described in the PaRIS international guidelines [10]. Some examples of the required general competencies are experience in conducting large-scale surveys, knowledge of sampling, survey data collection and quality control procedures and ability to access networks in the national primary care community. An example of the specific competencies is experience with and ability to communicate effectively with and engage with relevant national and regional stakeholders of the PaRIS survey in the country, including associations of citizens and patients and associations and networks of primary care professionals.

National project management teams in the participating countries had developed country roadmaps. They were responsible for gathering and providing information on national healthcare context, sampling frames, ethical review processes and survey approaches. The allocated consortium partners assisted the development of the country roadmaps by reviewing and revising them to meet the international guidelines. The development of country roadmaps was iterative (Table 1). The first drafts were developed by national project management teams, with the assistance of the consortium, based on international guidelines (see Additional material 1). The international PaRIS guidelines included six main activities for the development of the first draft of country roadmaps: (1) developing the first contact between national project management teams and consortium; (2) collecting information on the country healthcare context; (3) involving stakeholders and policymakers; (4) developing guidelines for sampling and recruitment of primary care practices and patients; (5) obtaining ethical approval for the survey; (6) establishing the country roadmap.

**Table 1:** List of activities to develop country roadmaps including the section on stakeholder engagement (chronological order)

Activity	Responsible and contributions
Development of survey manuals that provide a structured outline for country roadmaps, including primary care professional engagement (Additional material 1)	Consortium with the feedback of the OECD
Development of written country roadmaps for field trial that provide contextual information as well as implementation plans in relation to sampling, data collection, and stakeholder engagement	National Project Management teams with the assistance of Consortium and the feedback of the OECD
Preliminary analysis of stakeholder engagement activities in 17 country roadmaps for field trial	OECD and Consortium with the input of representatives from primary care professional organisations, namely, WONCA
Dissemination of results and sharing good practice examples from field trial [11]	OECD and Consortium with the input of representatives from primary care professional organisations, namely, WONCA
90-min workshop with national project management teams to provide feedback and discuss the findings [11]	OECD and Consortium
New guidance document on stakeholder engagement, incorporated in survey manuals (Additional material 1)	OECD with the feedback of Consortium
Development of final country roadmaps for main survey that provide contextual information as well as implementation plans in relation to sampling, data collection, and stakeholder engagement	National Project Management teams with the assistance of Consortium and the feedback of the OECD

The first drafts were then reviewed independently by at least two OECD staff working on the PaRIS project and revised by National Project Management teams with the feedback received. The type of feedback concerning the engagement section included general recommendations, such as the need to increase efforts to engage with primary care professionals or specific recommendations such as the establishment of an advisory board consisting of primary care professionals and other key stakeholders. The revised country roadmaps were then reviewed again and approved by the OECD Secretariat if they met the international guidelines. Countries needed the approval from the OECD before data collection could start.

The development of stakeholder engagement in international activities had short, medium and long-term outcomes. While the expected short-term outcomes were to increase the national project managers’ understanding of and motivation for stakeholder engagement, the long-term outcome was to facilitate the establishment and consolidation of a culture of stakeholder engagement in health policy and research.

### Analysis

Two researchers (CK and JB) developed a standardized data abstraction form. Three researchers (MvdB, JMV, and NK) independently reviewed the form and provided feedback. The final version of the data abstraction form consisted of country name, timeframe of planned engagement activities, intended target primary care professional, communication channel of engagement, level of engagement, and purpose of engagement. The word count of the country roadmap including annexes and the section on engagement and dissemination activities were noted.

*The timeframe* described the study phase (e.g., development of country roadmaps, sampling, data collection) when the activity was planned to take place. *Intended target professionals* were coded as individual family doctors (which includes medical doctors who provide primary care services to the general population, i.e., family doctors/general practitioners), individual nurses, other individual primary care professionals, family doctor organisations, primary care professional organisations, nurse associations and others. Details of other categories were recorded as open text. *Communication channels* were coded as they were stated in the country roadmaps, such as group meetings (in person or online), written feedback, emails, social media and posters. *Engagement levels* were coded in three groups: codesigned, involved and informed/consulted. If primary care professionals were planned to be engaged throughout the implementation process and were partners in the decision-making role, this was coded as “co-designed”. If they were planned to be regularly engaged in certain activities (e.g., sampling or recruitment of respondents) but had a less active role in the overall implementation process, these activities were coded as “involved”. If they were planned to be engaged irregularly to gather their opinion on a specific activity without any commitment for incorporating them or they were informed of any activities for further distribution among their networks, these activities were coded as “informed/consulted”. *The purpose of the engagement* was coded as free text as stated in the country roadmaps.

Two researchers (CK and JB) extracted data from 17 finalized country roadmaps independently. The three steps of qualitative documentary analysis were followed: skimming, reading and interpretation [13]. To identify engagement activities, country roadmaps were read with a specific focus given to the communication, engagement, and dissemination section. Initially, two researchers (CK and JB) pilot-tested three random country roadmaps to test the data extraction. Based on the discussions on these three country roadmaps, small clarifications were made to the data extraction. For example, local or regional health authorities were excluded from coding, and the details of other primary care professionals were collected in a separate column. Then, the rest of the country roadmaps were coded by considering these changes. There were 129 and 133 entries, respectively, by CK and JB in the first round of coding. Both researchers reviewed the initial coding, and any disagreement was resolved through joint review of the codes and discussion between CK and JB. The final set included 129 entries concerning planned engagement activities.

Data collection and analysis were iterative and characterised by evolved results, meaning that findings continually informed whether and how to obtain and interpret data [14]. For example, based on the information provided on the sequence of activities and engagement activities and their expected outcomes, the researchers (CK and JB) interpreted and coded the expected starting time of the engagement plans. A content analysis was performed by classifying and summarizing the data.

Quotes are anonymised to present the results. A purposeful sample was chosen by CK and JB based on relevance and fitness to serve for illustration. The choice of quotes was reviewed by (MB, OG, FG, AR, JMV, NK) and some were changed to enhance the illustration and, to a lesser extent assure some spread in the origin of quotes by countries and activities. Authors had two rounds of consultation to select the final series of quotes used to describe the results.

## Results

The length of country roadmaps varied across countries from 3719 words in Norway to 12 534 words in Australia (Table 2). There were several reasons for this variation. For example, some national project managers provided more detailed background information about their country’s context than others. In some cases, the healthcare system and/or primary care system was very complex and needed some explanation around the implementation. For some countries, small deviations from the international study design (e.g., modes of data collection via online survey, telephone survey or face-to-face interview) required more detail on the reasons and anticipated consequences of deviations. The length of the section on engagement activities also varied from 318 words in Canada to 3884 words in Australia. Similar reasons to the length of country roadmaps explained the variations. In terms of the proportion of engagement activities in the country roadmaps, the share of sections on engagement varied from 5% in the Czechia and Spain to 31% in Australia, displaying a median of 11% (IQR = 8–13%). On the basis of content analysis, we generated six main themes, presented in Table 3 with examples of quotes. In the following section, we describe the themes with quotes from country roadmaps.

**Table 2:** Key descriptions of countries and their country roadmaps

Country	Length of country roadmap (words)	Length of communication, engagement, and dissemination section (words)	Proportion of engagement activities in the document (%)
Australia	12 534	3884	31
Belgium	8425	827	10
Canada	4672	318	7
Czechia	8676	433	5
France	8174	1073	13
Greece	6270	712	11
Iceland	5527	418	8
Italy	6608	1506	23
Luxembourg	10 552	1171	11
Netherlands	8489	1062	13
Norway	3719	778	21
Portugal	5713	798	14
Romania	7097	698	10
Saudi Arabia	7012	531	8
Slovenia	6138	496	8
Spain	11 158	591	5
Wales (UK)	7866	1107	14

**Table 3:** Summary table of themes and examples of quotes

Themes		Selected quotes
**Building foundations of engagement:** setting purpose		“The plan is for the different stakeholders to develop the study in collaboration, supported by an international consortium, i.e., the PaRIS-SUR consortium, and the OECD Secretariat. By making this a shared undertaking, policy makers, patients and health care providers are involved to ensure that instruments and indicators are relevant for them.” *Country 12*
**Defining the target:** engaging different primary care professional groups		“Individual meetings will be planned with each organisation to inform and consult them on methodological items but also to create a collaboration and a support in the GPs recruitment through their communication channels (as newsletters, website and social media).” *Country 2*
**The timing: **engaging at different phases of the survey		*"We will then approach these (authors: national primary care professional) medical associations and demand their cooperation to spread information about the PaRIS project on their websites before the starting of the main trial, to support the project among GPs (authors: abbreviation for general practitioners), and to encourage their GP members to participate in the (authors: modified name of the PaRIS survey in the national context) survey.” Country 9*
**Reaching out: **using different communication channels to engage		*“The following awareness materials will be developed for use and dissemination in (Country name) in (Country languages): A webpage …; Information leaflets and pamphlets for patients; Frequently Asked Questions (FAQs) sheet for both patients and providers; Academic research articles about the PaRIS project in local and international scientific journals; Banners and posters distributed and arranged in hospitals and PHC centres; Awareness video to be posted on social media.” Country 14*
**Meaningful engagement:** choosing the level of engagement	Co-designed	*“The Ministry of Health appointed a working group for PaRIS. The working group consists of representatives from the institutions participating in the project: (Name) Community Health Centre, (Name) Department of Family Medicine, National Institute for Public Health, Ministry of Health.” Country 15*
	Involved	*“The PaRIS project has its own national advisory committee in (Country name), consisting of GPs (authors: abbreviation for general practitioners), representatives from the government and patient organisations.” Country 11*
	Informed/consulted	*“Various information channels will be used to enhance participation and overall visibility of project among primary healthcare providers.” Country 7*
**Anticipated challenges and facilitators for engagement**		*“It is likely that the (national family doctor organisation name) will have some concerns about the possible additional burden on GPs (authors: abbreviation for general practitioner), and risks associated with consolidating comparable data, including the potential for performance assessment across practices or to use the information for commissioning of services.” Country 1*

### Building foundations of engagement: setting the purpose

National project managers mentioned that increasing the relevance of the survey and its findings in the national context, gathering opinions, ensuring successful implementation of the PaRIS survey in the country’s context, and improving response rates were the main purposes of engagement activities.

*“The plan is for the different stakeholders to develop the study in collaboration, supported by an international consortium,* i.e.,* the PaRIS-SUR consortium, and the OECD Secretariat. By making this a shared undertaking, policy makers, patients and health care providers are involved to ensure that instruments and indicators are relevant for them.” Country 12*

*“A Steering Committee was set up at the beginning of the project, gathering the main national GPs (authors: abbreviation for general practitioners) and patient organisations and research institutes. The purpose is to codesign the implementation methodologies with the expertise of each other.” Country 2*

While there were national project managers who explicitly mentioned the purposes of primary care professional engagement (e.g., to encourage family doctors to participate in the survey), the purpose was not systematically reported in all country roadmaps.

### Defining the target: engaging different primary care professional groups

The primary care professionals that national teams aimed to target were family doctor organisations, individual family doctors, primary care organisations and community nurse associations. A few countries also planned to engage Practice-Based Research Networks in primary care.

The main reasons for engaging family doctor organisations were to co-develop the survey in the national context and/or to reach out to their members. Some countries asked professional organisations to provide a support letter for the survey to be sent to individual family doctors during the recruitment phase.

*“Individual meetings will be planned with each organisation to inform and consult them on methodological items but also to create a collaboration and a support in the GPs recruitment through their communication channels (as newsletters, website and social media).” Country 2*

Family doctors were the target group in all countries, as they were the respondents to the provider questionnaire. A few countries targeted a broader professional group addressing all primary care professionals (individual professionals not specified). One country specifically mentioned community nurse associations as one of the main target groups for engagement.

*“Additionally, meetings with scientific and patient organisations are also organised to collect and discuss improvement initiatives and to disseminate the project. The participating organisations are the following: Scientific Medical Association: Organisation of Family and Community Medicine; Scientific Nursing Association: Community Nursing and Primary Care Associations” Country 16*

Approximately one-third of countries appoint a family doctor or a nurse as National Project Manager. Among them, there were family doctors or nurses who were still practicing in a primary care facility (community health centre) and had an academic affiliation at a university (department of family medicine and primary care).

*“The Ministry of Health appointed a working group for PaRIS. This working group consists of representatives from the institutions participating in the project: (Name) Community Health Centre (leading institution), (Name) Department of Family Medicine, National Institute for Public Health, and Ministry of Health..” Country 4*

### The timing: engagement at different phases of the survey

The timing of engagement activities in the research process was not clearly stated in the country roadmaps. Most project management teams planned to engage primary care professionals throughout the project as part of the co-development process.

*"We will then approach these (authors: national primary care professional) medical associations and demand their cooperation to spread information about the PaRIS project on their websites before the starting of the main trial, to support the project among GPs (authors: abbreviation for general practitioners) and to encourage GP members to participate in the (authors: modified name of the PaRIS survey in the national context) survey.” Country 9*

*“The (Organisation) has established the PaRIS Governance Committee to provide strategic advice and support to roll out and embed the survey across the (Country name) primary care system. Membership of the Committee includes key representatives from the primary care sector.” Country 1*

The engagement of individual primary care professionals was usually planned before data collection (during the sampling or recruitment phase) or during data collection. The aim was to improve response rates for the PaRIS provider questionnaire.

### Reaching out: using different communication channels to engage

Countries planned to use various communication channels, ranging from in-person meetings to social media campaigns. One of the most common methods was organizing online meetings or webinars. The frequency of meetings was not always indicated. Some countries mentioned 3–4 times per year or irregular meetings on a need basis with their advisory groups. The length of these meetings was not specified in the country roadmaps.

*“We have constituted a committee of stakeholders. The meeting took place in March 2022. This meeting had several purposes: …. We have organised a new meeting of the same committee in the beginning of May (2023). As most methodological issues were decided at that moment, the meeting was mostly dedicated to presenting a follow-up on the survey, the questionnaires, and ask for recommendations on what themes the data analysis should focus on.” Country 5*

To inform individual primary care professionals, almost all countries planned to prepare written materials such as brochures, posters, or emails. Several countries planned to publish articles in national scientific journals to raise awareness about the survey and improve response rates of primary care professionals. Using emailing lists of professional organisations or health authorities was a common method to distribute promotion materials. Promotion via social media was another common method to raise awareness about the survey and improve response rates. Project managers also developed a dedicated website/webpage to inform primary care professionals about the survey and answer frequently asked questions. In countries with more than one official language, the promotion materials were developed in all official languages.

*“The following awareness materials will be developed for use and dissemination in (Country name) in (Country languages): A webpage …; Information leaflets and pamphlets for patients; Frequently Asked Questions (FAQs) sheet for both patients and providers; Academic research articles about the PaRIS project in local and international scientific journals; Banners and posters distributed and arranged in hospitals and PHC centres; Awareness video to be posted on social media.” Country 14*

All but one country planned to disseminate the results of the PaRIS survey to primary care professionals who responded to the questionnaire. A few of them planned to develop online dashboards, and a few planned to organise broader events with stakeholders, including primary care professionals, to discuss the results and translate them into policy.

*“We will share key findings from the main trial of the PaRIS survey with participants who wish to receive them. Accordingly, we will send an accessible summary of the results of the main trial to all participating GP (abbreviation for general practitioner) practices. … Survey findings will also be disseminated by presenting the results in the regular meetings/seminars of the GP (abbreviation for general practitioner) associations, as well as patient association.” Country 9*

### Meaningful engagement: choosing the level of engagement

The engagement of primary care professionals was planned at different levels in the participating countries. More than half of the countries had at least one engagement plan for co-designing the PaRIS survey in their national contexts. In the co-design process, representatives of family doctor organisations or other primary care organisations were the main invitees to national project management teams or governing bodies. In almost all countries, family doctors or other primary care professional representatives were involved in the process through advisory boards that did not have a decision-making role. Only in one country there were no plans for either co-design or involvement; the engagement of primary care professionals was planned as informing or consulting them when needed.

*“The Ministry of Health appointed a working group for PaRIS. The working group consists of representatives from the institutions participating in the project: (Name) Community Health Centre, (Name) Department of Family Medicine, National Institute for Public Health, Ministry of Health.” Country 15*

*“The PaRIS project has its own national advisory committee in (Country name), consisting of GPs (authors: abbreviation for General Practitioners), representatives from the government and patient organisations.” Country 11*

*“Various information channels will be used to enhance participation and overall visibility of project among primary healthcare providers.” Country 7*

### Anticipated challenges and facilitators of engagement

A few countries mentioned some anticipated challenges in engaging with primary care professionals. These were high burden in terms of workload on time-poor family doctors, concerns that providers would be publicly benchmarked, the survey being too focused on family doctors and increased workload on top of baseline workload levels referred to earlier due to the COVID-19 pandemic leaving less space for other additional activities. One country mentioned that including primary care champions was an effective engagement strategy to increase response rates. However, due to limited funding of the project, the national project management team decided to not implement this option.

*“It is likely that the (national family doctor organisation name) will have some concerns about the possible additional burden on GPs (authors: abbreviation for general practitioner), and risks associated with consolidating comparable data, including the potential for performance assessment across practices or to use the information for commissioning of services.” Country 1*

A few countries mentioned that they had already experienced engagement of primary care professionals in other national surveys. Those project managers perceived their previous experience as a facilitator for primary care professional engagement in the PaRIS survey. Some countries mentioned ongoing relationships or establishing long-term engagement with representatives of family doctors or primary care professional organisations.

Planning and scheduling of regular national cross-sectional surveys, embedding patient-reported measures in accreditation and continuous professional development programs were reported as means for long-term engagement with primary care professionals.

*“The medium- to long-term objective for (country name) is that patient-reported measures are collected routinely by participating in general practices as part of usual care processes, with data being invited from all relevant patients to inform clinical decision-making. … The momentum for this kind of change (authors: periodic cross-sectional survey of random sample of patients) will require active support by a motivated community of GP (authors: general practitioner) practices. There may also be scope to encourage greater participation by other means, for example, through general practice accreditation and/or continuing professional development. (Country name) is exploring these options with (national family doctor organisation name).” Country 1*

## Discussion

This study aimed to assess the plans for engaging primary care professionals in an international survey in 17 countries—the PaRIS survey. We found that the main purposes of assessing plans of engagement were to increase the relevance of the end-point survey results to improving the clinical practice of primary care professionals and/or improving response rates for the survey. Among primary care professionals, family doctor organisations and individual family doctors were most often targeted for engagement activities, as compared to nurses or allied health professionals. There were large variations in engagement levels across countries, with approximately half of countries planning to co-develop the PaRIS survey with primary care professionals. A variety of communication channels were used, depending on the objective of the engagement, mostly group meetings (in-person or online) and electronic or printed promotional materials such as brochures.

### Comparison with literature

Increasing the relevance of the survey in the national context, gathering views and improving response rates were the main overall purposes of primary care professional engagement activities in the national implementation plans. Previous studies reported similar purposes for professional engagement [4, 6, 7]. Engagement of healthcare professionals has also been shown to improve the acceptability of routine collection and use of patient-reported measures in clinical practice and implementation of patient-reported measures [15–17]. Healthcare professionals consider the advantages and disadvantages of such data collection and use, its appropriateness for the context of their clinical practice setting and their intention to collect and use it in the long term [16]. To succeed, developing a common understanding of the engagement activity, its importance and expected outcomes are key points to include in engagement plans [4, 18]. In this study, we focused on exploring the plans developed by national project managers; therefore, details on the perspectives of professionals and professional organisations were not reported.

Most countries we studied decided to focus their efforts on family doctors in their national implementation plan. Although family doctors are the main point of contact for the care of people with chronic conditions in most countries, the PaRIS provider questionnaire was carefully designed to be completed by any primary care professional or allied professional, and the sampling unit was a primary care facility. Diversity in the organisation of primary care services across countries and variations in the tasks and roles of nurses and other primary care professionals in the countries may explain this result [19, 20]. Given the increasing workload of family doctors, the increased survey burden on them and the lack of protected time for research activities, engaging other primary care professionals might increase participation in the survey by sharing tasks, thereby increasing response rates [21–23]. Advancing the use of digital tools such as searchable electronic health records to collect data, particularly systems that use electronic data capture, might be helpful in overcoming time and workload challenges [4] and allow for professionals’ participation to focus more on the development of survey tools and implementation of recommendations than on data collection itself.

We found that most countries planned to engage professional general practice/family medicine organisations as a first step in the engagement of professionals, and this was perceived as a facilitator by national project management teams. Previous research concurs that the involvement of leadership (such as professional organisations) and existing relationships with professional organisations appear to be key factors in ensuring the engagement of individual professionals [4, 18, 24]. The support of international umbrella organisations can help with identifying and connecting national organisations, reduce the resources needed for streamlining engagement and create a culture of engagement at national level.

Boosted by the rigorous support and advice on engagement activities at the international level, all countries engaged with primary care professionals. However, we found that national project management teams used different methods of collaboration to link with different groups, and only a few countries actually proposed co-designing the survey with healthcare professionals to include their perspectives. The importance of meaningful engagement is highlighted in the literature. Though the topic is relatively new in international literature, frameworks have been developed to improve the inclusion of perspectives of stakeholders [4, 11, 18, 25]. To ensure a consistent strategy in international studies, a guidance document on stakeholder engagement as recommended by the international PaRIS project management team can help with identifying key primary care professional stakeholder groups and planning meaningful engagement activities with each group in the participating countries/entities. Following the initial guidance, participating countries may vary slightly in their engagement activities.

In our study, one national project management team mentioned limited resources as a barrier to primary care professional engagement at the national level. Previous research showed that lack of resources including time needed to engage stakeholders in the timeframe of research grants were perceived as barriers by project managers [8]. It is necessary to allocate a part of the resources to engagement work and to clearly define what the engagement looks like with each stakeholder group, what the activity entails, and what is the expected time and resource investment needed for the activity. International collaboration and learning can help reduce the efforts required to engage primary care professionals by providing guidance and sharing good practice examples.

### Strengths and limitations

Our study describes several dimensions of engagement with primary care professionals, including engagement levels, and fills a significant gap in the literature. Another strength of the study is the collaboration of researchers from across the OECD, an international consortium, and representatives from primary care professional organisations, which provided a wide range of interdisciplinary perspectives on the engagement of primary care professionals.

One of the limitations of this study was the use of written country roadmaps as the source of information. These documents detailed the engagement plans in the countries, and we did not have information on whether these activities were realized, if realized, to what extent, and what the outcomes of the engagement activities were. This work can be extended in the future to explore how the characteristics of the country roadmaps predicted the actual implementation of planned activities and reached the outcomes according to the logic model. In addition, we analysed the plans of national project management teams. We do not have information about the perspectives and motivation of primary care professionals in participating countries. Further work can explore the perspectives of primary care professionals on engagement in international research activities.

### Implications for practice and policy

International collaboration and guidance can reduce the time and resources allocated by national project management teams for stakeholder engagement activities. Sharing good practices can help address fundamental challenges of engagement activities and promote good practice examples. However, as one size does not fit all, national project management teams will need to adapt and co-develop their engagement strategies. Engagement requires significant resources. It should therefore be included in planning and budgeting. Treating engagement activities as an afterthought or closing entry does not do justice to their importance for the success and relevance of the project.

Due to differences in health system resources and structures, and variations within and between countries, it is important to establish systems for coordinating systematic engagement of primary care professionals at national level. Primary care professionals are trained to deliver person-centred care to individuals in the context of their families and local communities. Their national organisations can help to orientate them to the fact that their collective data has the potential to influence redesign and reallocation of national health system resources to improve their workloads. The oversight of international organisations has the potential to save time and human resources at both national and frontline practice level.

Key professional organisations could provide insight into the clinical context while ensuring the relevance of data to both clinical practice and the national healthcare systems. To achieve better outcomes, investing in long-term relationships with key representatives of professional organisations has potential to be strategically helpful in health research and policy.

The participating countries and stakeholders in this study designed and adapted the PaRIS survey. Building a large-scale international survey has involved administrative, logistical, legal and practical challenges that require time, resources and energy. The first cycle of the PaRIS survey has provided valuable lessons and examples of good practices for establishing infrastructures in the participating countries. In the next steps of the PaRIS survey, countries or stakeholders could further build on these initial experiences for future data collections and integrate the PaRIS survey tools into national frameworks in several ways such as part of professional accreditation and continuous professional development. Meaningful engagement of primary care professionals in the countries will also be essential in these next steps to inform health policy.

## Conclusion

Healthcare is constantly evolving, so the engagement of healthcare professionals in health research and policy is crucial to drive evidence-informed policy and practice. In this large-scale international study, we provided international leadership, guidance and support to national teams facilitating the engagement of primary care professionals in the PaRIS survey. During this process, we observed differences between countries in execution of engagement plans in national contexts. Ongoing collaborative research activities at the international and national levels can foster a culture of engagement with primary care organisations and individual clinical and academic professionals. This collaborative approach can enhance meaningful engagement and optimise the potential for improving healthcare systems, in line with the emerging and evolving needs of stakeholders.

### Supplementary Information

Additional file 1. International guidelines on primary care professional engagement and stakeholder engagement as part of PaRIS survey operations manual for the Main Survey.

## Data Availability

The datasets used and analysed during the current study are available from the corresponding author on reasonable request.

## Abbreviations

OECD: Organisation for Economic Co-operation and Development
PaRIS: Patient Reported Indicator Surveys
PREM: Patient reported experience measure
PROM: Patient reported outcome measure
WONCA: World Organisation of Family Doctors
