# Supplementary material for: Engaging primary care professionals in OECD’s international PaRIS survey: a documentary analysis
Source: Health Res Policy Syst. 2024 Jul 4;22:76. doi: 10.1186/s12961-024-01170-2 (PMC11223287; doi:10.1186/s12961-024-01170-2)
Supplement: Supplementary file 1 — Additional file 1. International guidelines on primary care professional engagement and stakeholder engagement as part of PaRIS survey operations manual for the Main Survey. [file 12961_2024_1170_MOESM1_ESM.docx]

# Supplementary material 1. International guidelines on primary care professional engagement and stakeholder engagement as part of PaRIS survey operations manual for the Main Survey

## International guidelines on primary care professional engagement as part of PaRIS survey operations manual for the Main Survey

## Note: This document was shared with national project management teams during the development phase, before the field trial and main survey data collections.

### Recommended approach to the primary care (PC) practice tasks

#### Introduction

This chapter sets out the recommended approach for delivering the PC practice tasks for the Main Survey. As detailed in chapter 4, it is essential that the patient data can be linked to the PC practice data, and PC practice involvement will be critical in achieving this. The tasks that will be undertaken by PC practices are:

- Facilitate drawing a sample of their patients either by completing the selection, or allowing a field worker or member of the NPM team to do so;
- Providing information about the representativeness of the patient sample; and,
- Completing the PC practice questionnaire, in order to make sure that PC practice data can be linked and provide context to the patient data.

NPMs will need to work closely with PC practices to achieve these tasks. It is essential for the success of the Main Survey that each of the participating PC practices complete all tasks! If there is a method of gaining patient samples from a central database which does not include practice involvement, NPMs should discuss this with their Consortium partner. As most countries will not have such a method available, the remainder of this chapter is focused on first sampling and recruiting PC practices, to then sampling patients from their records.

NPMs will need to support PC practices through the project to ensure they are willing and able to participate in the practice survey *and* to facilitate the participation of patients in the patient survey. The key steps that NPMs will need to undertake in supporting the PC practices are:

1. Developing the PC practice engagement and contact strategy, including strategies for wider

stakeholder engagement;

2. Sampling a representative sample of PC practices;

3. Recruiting participants from the sample of PC practices;

4. Briefing and supporting PC practices on how to select and compile representative patient

samples, or completing the patient selection process on behalf of the practices;

5. Transfer of patient sample files (if applicable);

6. Setting up the PC practice online survey (if needed);

7. Administration of the PC practice online survey.

Step 1 - Communication and engagement

The first step that all NPMs should undertake is developing a communications and engagement strategy, as this will be a core element in maximising participation and cooperation in the practice survey. This strategy will need to target not only PC practices but also wider stakeholders, who maybe influential in encouraging participation. These communications and engagement activities should start in advance of recruitment, and continue throughout this phase to help inform and “warm up” potential participants.

Table 1 details potential engagement strategies for key stakeholders. NPMs are encouraged to use these strategies and consider other methods that have worked well in other studies. Details of the proposed engagement strategies should be included in the CRM.

Table 1: Overview of relevant communication and engagement strategies to increase awareness

and participation in the PaRIS survey for practices

| **Key activity / strategy** | **Key communication channels / tools** |
| --- | --- |
| Involving PC professional organisations such as national colleges to help raise awareness and engagement for participation in the survey among members of relevant organisations. | To promote and inform members about the survey via the organisations’ newsletter, webpages, articles in magazines, emails, leaflets, and social media. |
| Raise awareness and engagement for participation in the survey via academic conferences. | To promote and inform stakeholders about the survey via academic conferences using posters or oral presentations. |
| Involving relevant national and regional government bodies, e.g. Ministry/Department of Health) to help raise awareness and engagement for participation in the survey. | To promote and inform stakeholders about the survey via official newsletters, webpages,  leaflets, and social media. |

## International guidance on stakeholder engagement as part of PaRIS survey operations manual for the Main Survey

## Note: This document was shared with national project management teams before the main survey data collection took place, after the field-testing of the survey design and instruments in the countries.

### Involving stakeholders and policymakers

The involvement and support of stakeholders is crucial for the success of the PaRIS survey. Their support and involvement may be needed to carry out the data collection in an effective and reliable manner. In addition, and perhaps even more important, stakeholders will be using the results of the survey to inform policies with the potential to make health systems more responsive to patient needs. As such, the involvement of stakeholders is not only helpful for data collection but should also provide a platform for meaningful interpretation of the results and to translate these results into actions.

The methods for stakeholder engagement may differ according to the administrative and political structure of a country. A stakeholder analysis using the power/interest grid (Diagram 1) might PaRIS-SUR Survey Operations Manual for the Main Survey help NPMs in identifying key stakeholders in their respective countries and managing their meaningful participation in the PaRIS survey:

- Fully engage/co-develop with the ones who have high power and high interest
- Keep regular contacts with/Involve the ones who have high power but low interest
- Consult and inform those who have low power but high interest
- Monitor the ones who have low power and low interest.

Given the specific role of patients and practices as key stakeholders in the PaRIS survey, their active participation is highly encouraged in decision-making groups, steering groups or advisory groups.


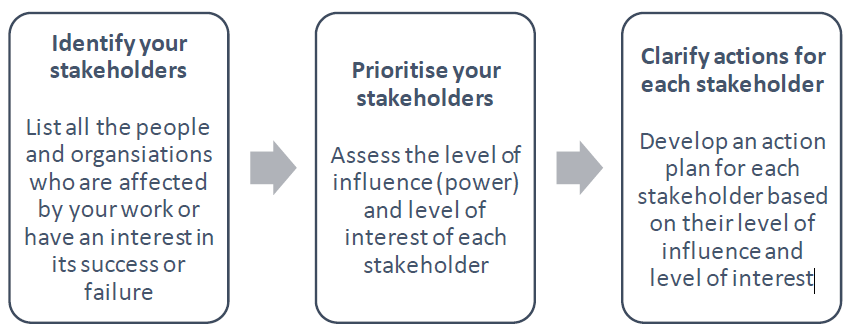


Diagram 1. Main steps in performing a stakeholder analysis using the power/interest grid

NPMs can provide insight in the most effective approach in their country. Therefore, they will be leading in identifying and conducting outreach to appropriate stakeholder groups. The Consortium will facilitate the exchange of experiences among NPMs.

WONCA member organisations

WONCA world has sent a letter of recommendation to all its members to encourage them to support the PaRIS survey. In most countries, these members are influential among primary care practices. A list of WONCA members per country can be found on the WONCA Website:

https://www.globalfamilydoctor.com/AboutWonca/Regions.aspx.
